# An overview of viral infections of the nervous system in the immunosuppressed

**DOI:** 10.1007/s00415-020-10265-z

**Published:** 2020-10-13

**Authors:** Peter G. E. Kennedy

**Affiliations:** Institute of Infection, Immunity and Inflammation, College of Medical, Veterinary and Life Sciences, University of Glasgow, Glasgow, G61 1QH Scotland UK

**Keywords:** Virus, Nervous system, Immunosuppression, Infection, PCR, Immunodeficiency

## Abstract

Several viruses have the capacity to cause serious infections of the nervous system in patients who are immunosuppressed. Individuals may be immunosuppressed because of primary inherited immunodeficiency, secondary immunodeficiency due to particular diseases such as malignancy, administration of immunosuppressant drugs or organ or bone marrow transplantation. The viruses capable of such opportunistic infection of the nervous system include herpes simplex virus (HSV), Varicella-Zoster virus (VZV), Cytomegalovirus (CMV), Epstein –Barr virus (EBV), Human Herpes virus type 6 (HHV-6), JC virus (JCV), enterovirus, measles virus and Covid-19. In most cases it seems likely that immunological defence mechanisms in the immunosuppressed are deficient which creates a suitable environment for certain viruses to become opportunistic in the nervous and other systems. Further research is required both to understand these opportunistic mechanisms in more detail and also to determine how many virus infections are modified by specific inborn errors of immunological responses.

## Introduction

An individual may become immunocompromised as a result of multiple factors. These include the administration of immunosuppressant drugs given for various medical conditions, an underlying disease such as Human Immunodeficiency Virus (HIV) infection or malignancy such as lymphoma which affects the immune system producing secondary immunodeficiency, various primary immunodeficiency syndromes such as severe combined immunodeficiency (SCID) with defects in T cells, B cells, and NK cells [1], or from organ or bone marrow transplantation. In practice, these factors are often present in the same patient so they do not act in isolation as in the case of a patient who receives immunosuppressant drugs to suppress organ rejection after bone marrow or organ transplantation. Immunosuppressant drugs include corticosteroids, chemotherapy for malignant diseases or for autoimmune and inflammatory conditions, targeted monoclonal antibody therapy, and drugs given as anti-rejection therapy [2, 3].

Viruses may reactivate under conditions of immunosuppression from various causes primarily because the immune mechanisms that normal suppress or limit viral replication become disrupted. In the situation of latent virus infection which is kept in check by the immune system the virus may reactivate and cause neurological disease. For example, in the case of Varicella Zoster (VZV) infection, following primary infection (chicken pox) the virus becomes latent in neurons in peripheral ganglia [4]. It may then reactivate causing herpes zoster (shingles), more commonly in conditions of immunosuppression, for instance in the elderly who have impaired cell mediated immunity (CMI) to VZV [5, 6]. Other conditions or drugs impairing CMI also increase the risk of VZV reactivation with possible neurological complications. A virus may more commonly infect individuals with waning or impaired immunity or it may cause a more severe infection, or it may do both.

Here I give an overview of the several viruses which have a propensity to replicate and/or reactivate under conditions of immunosuppression causing neurological disease. These include Herpes viruses including Herpes Simplex Virus type 1 and 2 (HSV-1, HSV-2), VZV, Cytomegalovirus (CMV), Epstein Barr virus (EBV) and Human Herpes Virus type 6 (HHV-6). Also included here is JC Virus, and, in brief, enteroviruses, measles and the recently recognised Covid-19 (SARS-CoV2) infection. In many cases there is included an indication of the Class of Evidence and Level of Recommendation for a therapeutic intervention or an investigative procedure, and these are based on previously published criteria which are also used for official European Academy of Neurology guidelines [7, 8, 9].

## Specific viral infections in the immunosuppressed

### Herpes simplex virus (HSV)

While HSV-1 is classically associated with herpes simplex encephalitis (HSE) [10], HSV-2 accounts for about 10% of cases of HSE and when it does so it is typically associated with immunosuppression [11] so is of greater relevance to the current overview. The typical clinical features of HSE are well recognised with predominant involvement of the fronto-temporal brain regions. It should also be mentioned that immunodeficiencies involving the Toll-like receptor 3 (TLR3) pathway are well established, at least in children, to predispose to HSE [12]. Children with this immune defect are also likely to have relapses of HSE [13]. Treatment of HSE due to both viruses is well established to be with intravenous acyclovir 30 mg/Kg/day for 10–14 days given as 10 mg/kg given as an intravenous infusion over one hour three times daily (Class 1), though in the immunosuppressed patient the duration of therapy should be 21 days [8] (Class IV). Further, after reactivation from lumbosacral ganglia where the virus may remain in a latent form for many years, HSV-2 can cause genital herpes and has been reported as a likely cause of lumbosacral radiculopathy [14] particularly in the immunosuppressed state. A diagnosis in both conditions can be achieved using cerebrospinal fluid (CSF) Polymerase Chain Reaction (PCR) to detect HSV-2 DNA (Class 1, Level A) [9] Treatment should again be with acyclovir. Mild or atypical HSVE may be associated with infection with either HSV-1 or HSV-2. Thus, a retrospective study using PCR analysis found that mild or atypical HSV encephalitis, due to either HSV-1 or HSV-2, was frequently associated with an immunocompromised state and they reported that an asymmetric HSV infection affecting predominantly the non-dominant temporal lobe was typical [15].

### Varicella-Zoster virus (VZV)

Reactivation of latent VZV from human ganglia is a well-recognised complication of immunsuppression resulting in a variety of neurological complications, due, as indicated above, to symptomatic impairment of cell-mediated immunity to the virus. Indeed VZV infections are among the most important of all viral infections that affect the central nervous system (CNS) and peripheral nervous system (PNS) in immunocompromised individuals. The protean manifestations of VZV-induced neurological disease have been described in detail elsewhere [6], but the main complications include herpes zoster (shingles) followed by post-herpetic neuralgia (PHN) in some cases the incidence of which rises with increasing age, VZV vasculopathy, myelitis, and *zoster sine herpete* where VZV reactivates in the absence of the typical zosteriform rash [16, 17, 18]. In chronic graft versus host disease following bone marrow transplantation VZV reactivation is also a recognised opportunistic viral infection that may mimic lymphoma [19]. Treatment should be given to immunocompromised patients with herpes zoster consisting of intravenous acyclovir (in adults 10–15 mg/Kg 8 hrly for 10–14 days and in children 500 mg/m^2^ for 7–10 days) (Class IV). The role of acylcovir in imunocompetent individuals below 50 years is less clear. In patients with VZV-induced myelitis and vasculopathy it has been suggested that treatment should be with intravenous acyclovir for 14 days in combination with oral prednisolone for 5–7 days but this is based on experience with only a small number of patients [20] (Class IV). The diagnosis of an opportunistic VZV infection, including *zoster sine herpete* can be obtained using PCR to detect VZV DNA in the CSF (Class III, Level C), though detection of anti-VZV IgG in the CSF has a higher sensitivity for correctly diagnosing VZV vasculitis than does PCR [21]. The importance of host immunogenetics in determining VZV infection of the nervous system has recently been demonstrated. Thus it was shown that defects in the DNA sensor RNA polymerase III (POL III) confer selective increased susceptibility to infection of the central nervous system (CNS) causing encephalitis [22, 23] and such hereditary immune defects may result in reduced production of Type 1 Interferon which reduces the host’s anti-viral capacity. The role of zoster vaccination for the immunocompromised patient is currently not established.

### Cytomegalovirus (CMV)

CMV infection has an increased incidence under conditions of immunosuppression, particularly in patients with HIV infection. The virus can affect both the CNS and the PNS. Encephalitis and ventriculitis are recognised as well as retinitis. In the PNS an acute lumbosacral polyradiculopathy is well recognised, may be painful, and is associated with a CSF pleocytosis in half the cases and the MRI shows enhancing lesions in the conus, cauda equina and lumbosacral nerve roots [14]. Also recognised are mononeuritis multiplex, and myeloradiculopathy, and all these conditions can be confirmed using CSF PCR for CMV DNA with over 90% sensitivity and specificity [9] (Class II, Level B). CMV reactivation is particularly associated with renal transplantation. Though Guillain–Barre syndrome (GBS) and CMV polyradiculopathy are known to be associated with CMV infection, these conditions occur primarily in immunocompetent individuals. Where GBS occurs established treatment is with intravenous immunoglobulin (Class 1, Level A). Antiviral therapy of CNS and PNS CMV infections is not very satisfactory with data from controlled antiviral trials in CMV-induced neurological disease lacking. Gangciclovir, foscarnet and cidofovir have all been used, with combination therapy (gangciclovir and foscarnet) reported as being more effective than monotherapy in the important induction phase of CMV encephalitis [8] (Class 4). Such treatment can be followed by monotherapy with either of these two drugs, with a total duration of 6 weeks treatment in immunosuppressed individuals. In the PNS conditions one option is to give intravenous gangciclovir as first line therapy for 14–21 days.

### Epstein-Barr virus (EBV)

EBV infection of the nervous system can occur in both immunocompetent and immunosuppressed individuals, with the latter at greater risk of this infection especially if they also have HIV infection giving an increased risk of primary CNS lymphoma [24]. Most of our information is based on case reports and serological data in immunocompetent individuals as such infections are relatively uncommon (so only Class IV evidence). The neurological conditions known to be associated with EBV include plexopathy or radiculoplexopthy, acute radiculits, acute autonomic neuropathy and cranial neuropathies [14]. A rare complication of solid organ transplantation is a primary CNS post-transplant lymphoproliferative disorder and is typically an EBV-induced lymphoma, the risk being dependent on the type of transplant and the degree of immunosuppression [25]. CSF PCR for EBV DNA has a high sensitivity and specificity between 97 and 100% [9] (Class IV, Level C). No antiviral agents have been shown to be effective so far against EBV infections.

### Human herpes virus type 6 (HHV-6)

HHV-6 is a human herpes virus that is capable of reactivation in patients who are immunosuppressed including transplant patients. Following bone marrow transplantation HHV-6 may rarely cause encephalitis, typically being an opportunistic infection occurring in the period between pancytopaenia and chronic graft versus host disease [19]. HHV-6 has also been implicated in encephalitis or meningoencephalitis in immunocompromised persons, including HIV-positive individuals [26]. Anti-viral therapy that has been used in HHV-6 encephalitis includes foscarnet (for both HHV-6A and HHV-6B variants) and gangciclovir as an alternative for encephalitis caused by the HHV-6 B variant [8].

### JC virus (JCV)

JCV-induced progressive multifocal encephalopathy (PML) is a demyelinating disease associated mainly with immunosuppression, especially AIDS (5% at post-mortem), organ transplants, leukaemia, and the Multiple Sclerosis drug Natalizumab (Tysabri) [27, 28, 29]. It is caused by the polyoma virus JCV infecting oligodendrocytes causing demyelination and multifocal neurological features such as motor, cognitive, visual deficits, ataxia, and seizures with a progressive course. The CSF is typically non-inflammatory and normal, JCV antibody detection is useful and CSF PCR for JCV DNA is now an established method of diagnosis though a definitive diagnosis of PML can be made by brain biopsy. MRI imaging shows varied features including confluent multifocal white matter hyperintensities on T2-weighted images, usually without enhancement on post-contrast T1 weighted images. Contrast enhancement is seen in the context of Natalizumab and other situations where there is an immune reconstitution inflammatory syndrome. The prognosis is very poor, and there is no clearly effective treatment. While the specificity of JCV PCR is very good at around 98.5–100%, the sensitivity is variable and much less reliable at around 50–82% (Class II, Level B).

## Other viruses that may be more serious in the immunosuppressed

### Enterovirus

While enteroviruses such as poliovirus, echoviruses and coxsackie viruses are known to infect the immunocompetent human host causing a range of diseases including those of the CNS such as encephalitis, they may also be significant in the immunocomprised individual. Diseases such as meningitis, myocarditis and myopathy can be more common in the immunocompromised or neonatal host [30]. Of particular relevance here is the occurrence of a chronic meningoencephalitis in immunocompromised patients, and in particular those suffering from X-linked agammaglobulinemia in whom this chronic CNS infection is the commonest cause of death [30]. The diagnosis may be established by Reverse transcription (RT)‐PCR in the CSF for enterovirus. While the CSF specificity of this assay appears to be high (92–100%), the sensitivity is less certain with reports varying between about 31–95% (Class II, Level B) [9].

### Measles virus

While measles virus infection, normally a fairly mild though certainly uncomfortable condition, has the capacity to be serious in the immunocompetent, it is typically a far more dangerous infection in the immunosuppressed. Measles may rarely be followed by an acute post-measles encephalitis, which is the most common CNS complication in the immunocompetent as well as a rare primary measles encephalitis. Immunodeficient children are susceptible to measles inclusion body encephalitis where the measles virus persists in the brain. The latter is a very serious infection with a mortality of 75% [31]. In a report of four children with presumed measles inclusion body encephalitis who were treated for lymphatic malignancies by immunosuppressive drugs it was found that epilepsia partialis continua was a prominent feature in three patients, and all cases were fatal [32]. In these latter children measles virus was isolated from the brain in one case and identified immunologically in another, and measles virus nucleocapsids identical to those seen in subacute sclerosing panencephalitis (SSPE), a rare and invariably fatal complication of measles virus infection, were also identified in three of these patients. Two of the patients had impaired cellular immunity [32] which presumably favours the persistence of measles virus in the brain. Clearly, measles virus is a very significant opportunistic infection in patents who are immunsuppressed.

### Covid-19

In the author’s view, it is relevant to mention this recently recognised infection in the current context. It is known that malignant disease predisposes to Covid-19 infection with 7.2% of 138 patients having malignant disease as a co-morbidity in 1 major study [33]. Neurological features are now well recognised in Covid-19 infection indicating that the virus is neurotropic and include encephalitis, meningitis, myalgia and myositis, acute cerebrovascular disease, anosmia, Guillain–Barre syndrome, post-infectious acute disseminated myelitis and brainstem encephalitis [34, 35]. Since the infection is typically more serious in the elderly in whom the immune system is weaker, in terms of decreased cell-mediated immunity compared with younger individuals, in this sense at least a compromised immune system could predispose to neurological features. Further, not only does lymphopaenia occur as the Covid-19 infection progresses [36], but a pre-existing lymphopaenia secondary to cancer chemotherapy itself predisposes to Covid-19 infection (37). Since neurological complications are usually part of a more severe type of disease it seems likely that immunosuppression of some kind likely increases the chances of such complications occurring. It remains to be seen whether there are specific inborn errors of immunity that might predispose to particular neurological features in this infection.
